# Bis(diallyl­benzimidazolium) tetra­bromidocuprate(II)

**DOI:** 10.1107/S1600536808023039

**Published:** 2008-07-26

**Authors:** Marian Mys’kiv, Evgeny Goreshnik

**Affiliations:** a Department of Inorganic Chemistry, Ivan Franko National University, Cyryla and Mefodia 6, L’viv, Ukraine; b Department of Inorganic Chemistry and Technology, Jožef Stefan Institute, Jamova 39, 1000 Ljubljana, Slovenia

## Abstract

The structure of the title ionic copper(II) compound, (C_13_H_15_N_2_)_2_[CuBr_4_], is built up of isolated 1,3-diallyl­benzimidazolium cations and [CuBr_4_]^2−^ anions which are inter­connected by electrostatic inter­actions. Differences in packing of the heterocyclic cores results in a different structure compared with earlier investigated chloride and bromide analogues.

## Related literature

For related literature, see: Goreshnik *et al.* (1999 ▶, 2000 ▶); Hathaway (1982 ▶).
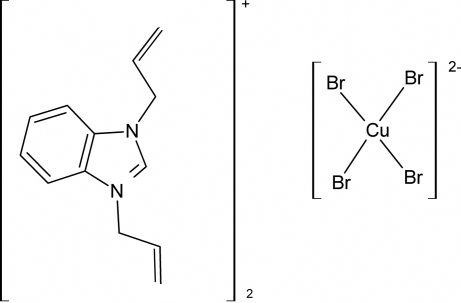


## Experimental

### 

#### Crystal data

(C_13_H_15_N_2_)_2_[CuBr_4_]
*M* *_r_* = 781.69
Monoclinic, 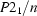

*a* = 10.8619 (7) Å
*b* = 15.3447 (7) Å
*c* = 18.3282 (10) Å
β = 105.451 (2)°
*V* = 2944.4 (3) Å^3^
*Z* = 4
Mo *K*α radiation
μ = 6.19 mm^−1^
*T* = 200 K
0.12 × 0.09 × 0.07 mm

#### Data collection

Rigaku Mercury CCD diffractometer
Absorption correction: multi-scan (Blessing, 1995 ▶) *T*_min_ = 0.522, *T*_max_ = 0.639
24051 measured reflections
6609 independent reflections
4956 reflections with *I* > 2σ(*I*)
*R*_int_ = 0.052

#### Refinement

*R*[*F* ^2^ > 2σ(*F* ^2^)] = 0.065
*wR*(*F* ^2^) = 0.136
*S* = 1.21
6609 reflections
316 parameters
H-atom parameters constrained
Δρ_max_ = 1.02 e Å^−3^
Δρ_min_ = −0.54 e Å^−3^

### 

Data collection: *CrystalClear* (Rigaku, 2001 ▶); cell refinement: *CrystalClear* data reduction: *CrystalClear*; program(s) used to solve structure: *SIR92* (Altomare *et al.*, 1993 ▶); program(s) used to refine structure: *SHELXL97* (Sheldrick, 2008 ▶); molecular graphics: *DIAMOND* (Brandenburg & Berndt, 1999 ▶); software used to prepare material for publication: *WinGX* (Farrugia, 1999 ▶) and *enCIFer* (Allen *et al.*, 2004 ▶).

## Supplementary Material

Crystal structure: contains datablocks I, global. DOI: 10.1107/S1600536808023039/dn2367sup1.cif

Structure factors: contains datablocks I. DOI: 10.1107/S1600536808023039/dn2367Isup2.hkl

Additional supplementary materials:  crystallographic information; 3D view; checkCIF report

## Figures and Tables

**Fig. 1.**
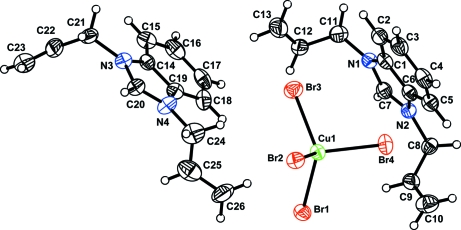
View of (I) (30% probability displacement ellipsoids)

**Fig. 2.**
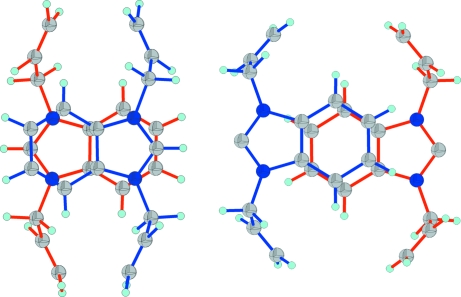
Difference in a packing of heterocyclic cores in [C13H15N2]+2[CuBr4]2- and [C13H15N2]+2[CuCl4]2- compounds

## supplementary crystallographic information

### Comment

The structure of the title compound (I) is built by isolated 1,3-diallylbenzimidazolium cations and CuBr_4_^2-^ anions which are interconnected by electrostatic interaction (Fig. 1). The copper(II) atom possesses a less common distorted tetrahedral coordination. The flattened tetrahedron of the Cu^II^ atom can be considered as a result of the Jahn–Teller effect similarly as it takes place in the structure of CsCuCl_3_ (Hathaway, 1982).

Compound I noticeably differs from earlier investigated chloride [C_13_H_15_N_2_]^+^_2_[Cu^II^Cl_4_]^2-^ (Goreshnik *et al.*, 1999) and chloride–bromide [C_13_H_15_N_2_]^+^_2_[CuCl_2.58_Br_1.42_]^2-^ (Goreshnik *et al.*, 2000) derivatives. Last two compounds are isostructural and crystallize, contrary to compound I, in an orthorhombic Fddd space group. The main difference between two structural types is the packing of the closest benimidazole rings. In chloride and chloride–bromide derivatives two closest heterocyclic cores are oriented in a 'head-to-tail' manner with the location of benzene ring of one organic molecule opposite the imidazole ring of another one (Fig. 2 left). The planes of the closest benzimidazole rings are slightly tilted. In compound I benzene ring of one organic moiety is oriented strictly opposite the benzene ring of another one (Fig. 2 right). Two closest benzimidazole cores appear to be strictly parallel, and the ring–ring distanse of 3.752 (9) Å indicates the presence of π–π stacking interaction.

### Experimental

Compound I was synthesized from Cu(CF_3_COO)_2_H_2_O and 1,3-diallylbezimidazolium bromide in ethanol solution.

### Refinement

All H atoms were fixed geometrically and treated as riding with C—H = 0.93 Å (aromatic) or 0.97 Å (methylene) with U_iso_(H) = 1.2U_eq_(C ).

### Crystal data

**Table:**

(C_13_H_15_N_2_)_2_[CuBr_4_]	*F*_000_ = 1532
*M**_r_* = 781.69	*D*_x_ = 1.763 Mg m^−^^3^
Monoclinic, *P*2_1_/*n*	Mo *K*α radiation λ = 0.71069 Å
Hall symbol: -P 2yn	Cell parameters from 8643 reflections
*a* = 10.8619 (7) Å	θ = 1.8–28.9º
*b* = 15.3447 (7) Å	µ = 6.19 mm^−^^1^
*c* = 18.3282 (10) Å	*T* = 200 K
β = 105.451 (2)º	Chunk, black
*V* = 2944.4 (3) Å^3^	0.12 × 0.09 × 0.07 mm
*Z* = 4	

### Data collection

**Table:**

Rigaku Mercury CCD diffractometer	*R*_int_ = 0.052
dtprofit.ref scans	θ_max_ = 29.5º
Absorption correction: multi-scan(Blessing, 1995)	θ_min_ = 1.8º
*T*_min_ = 0.522, *T*_max_ = 0.639	*h* = −15→15
24051 measured reflections	*k* = −21→21
6609 independent reflections	*l* = −25→25
4956 reflections with *I* > 2σ(*I*)	

### Refinement

**Table:**

Refinement on *F*^2^	Secondary atom site location: difference Fourier map
Least-squares matrix: full	Hydrogen site location: inferred from neighbouring sites
*R*[*F*^2^ > 2σ(*F*^2^)] = 0.065	H-atom parameters constrained
*wR*(*F*^2^) = 0.136	*w* = 1/[σ^2^(*F*_o_^2^) + (0.0337*P*)^2^ + 3.822*P*] where *P* = (*F*_o_^2^ + 2*F*_c_^2^)/3
*S* = 1.21	(Δ/σ)_max_ < 0.001
6609 reflections	Δρ_max_ = 1.03 e Å^−^^3^
316 parameters	Δρ_min_ = −0.54 e Å^−^^3^
Primary atom site location: structure-invariant direct methods	Extinction correction: none

### Special details

**Table:**

Geometry. All e.s.d.'s (except the e.s.d. in the dihedral angle between two l.s. planes) are estimated using the full covariance matrix. The cell e.s.d.'s are taken into account individually in the estimation of e.s.d.'s in distances, angles and torsion angles; correlations between e.s.d.'s in cell parameters are only used when they are defined by crystal symmetry. An approximate (isotropic) treatment of cell e.s.d.'s is used for estimating e.s.d.'s involving l.s. planes.

### Fractional atomic coordinates and isotropic or equivalent isotropic displacement parameters (Å^2^)

**Table:**

	*x*	*y*	*z*	*U*_iso_*/*U*_eq_	
Br1	−0.00902 (7)	0.69985 (4)	0.39870 (4)	0.0588 (2)	
Br2	0.08214 (7)	0.78208 (5)	0.22471 (4)	0.05540 (19)	
Br3	0.28719 (7)	0.88041 (5)	0.39572 (4)	0.0670 (2)	
Br4	−0.04726 (7)	0.93993 (5)	0.37629 (4)	0.0606 (2)	
Cu1	0.08067 (7)	0.82226 (5)	0.34949 (4)	0.0479 (2)	
N1	0.1331 (5)	1.0593 (3)	0.1875 (3)	0.0506 (13)	
N2	−0.0731 (5)	1.0430 (3)	0.1659 (3)	0.0450 (12)	
N3	0.6672 (5)	0.6752 (3)	0.1157 (3)	0.0500 (13)	
N4	0.4599 (5)	0.6754 (4)	0.0790 (3)	0.0596 (15)	
C1	0.0793 (7)	1.0878 (4)	0.1133 (4)	0.0498 (15)	
C2	0.1342 (7)	1.1211 (4)	0.0586 (4)	0.0595 (18)	
H2	0.2218	1.1294	0.0679	0.071*	
C3	0.0514 (8)	1.1407 (4)	−0.0092 (4)	0.0654 (19)	
H3	0.0841	1.1630	−0.0473	0.078*	
C4	−0.0790 (8)	1.1290 (4)	−0.0241 (4)	0.0633 (19)	
H4	−0.1306	1.1428	−0.0719	0.076*	
C5	−0.1354 (7)	1.0974 (4)	0.0303 (4)	0.0578 (17)	
H5	−0.2232	1.0906	0.0211	0.069*	
C6	−0.0509 (6)	1.0766 (4)	0.0994 (3)	0.0472 (14)	
C7	0.0392 (6)	1.0332 (4)	0.2152 (4)	0.0501 (15)	
H7	0.0507	1.0106	0.2636	0.060*	
C8	−0.1982 (6)	1.0191 (4)	0.1768 (4)	0.0524 (15)	
H8A	−0.2591	1.0653	0.1573	0.063*	
H8B	−0.1908	1.0128	0.2304	0.063*	
C9	−0.2457 (7)	0.9362 (4)	0.1373 (4)	0.0599 (17)	
H9	−0.2002	0.8855	0.1547	0.072*	
C10	−0.3459 (8)	0.9295 (6)	0.0805 (5)	0.084 (3)	
H10A	−0.3936	0.9789	0.0618	0.101*	
H10B	−0.3702	0.8755	0.0585	0.101*	
C11	0.2709 (7)	1.0506 (5)	0.2243 (4)	0.0643 (18)	
H11A	0.2847	1.0497	0.2788	0.077*	
H11B	0.3159	1.1005	0.2115	0.077*	
C12	0.3226 (7)	0.9698 (5)	0.2000 (4)	0.0636 (18)	
H12	0.2953	0.9167	0.2144	0.076*	
C13	0.4048 (8)	0.9693 (5)	0.1592 (5)	0.090 (3)	
H13A	0.4336	1.0216	0.1440	0.108*	
H13B	0.4345	0.9166	0.1454	0.108*	
C14	0.6251 (6)	0.7068 (4)	0.1764 (3)	0.0474 (14)	
C15	0.6927 (7)	0.7363 (4)	0.2464 (4)	0.0602 (18)	
H15	0.7815	0.7380	0.2613	0.072*	
C16	0.6190 (9)	0.7634 (5)	0.2931 (4)	0.070 (2)	
H16	0.6601	0.7836	0.3413	0.085*	
C17	0.4853 (9)	0.7619 (4)	0.2712 (4)	0.064 (2)	
H17	0.4406	0.7801	0.3052	0.077*	
C18	0.4193 (7)	0.7341 (4)	0.2007 (4)	0.0624 (19)	
H18	0.3305	0.7338	0.1856	0.075*	
C19	0.4916 (7)	0.7064 (4)	0.1530 (4)	0.0526 (16)	
C20	0.5660 (6)	0.6565 (4)	0.0598 (3)	0.0524 (15)	
H20	0.5688	0.6333	0.0134	0.063*	
C21	0.7995 (6)	0.6605 (5)	0.1136 (4)	0.0594 (17)	
H21A	0.8490	0.7126	0.1315	0.071*	
H21B	0.8010	0.6510	0.0615	0.071*	
C22	0.8619 (8)	0.5839 (6)	0.1607 (6)	0.081 (2)	
H22	0.8817	0.5904	0.2130	0.098*	
C23	0.8892 (9)	0.5139 (7)	0.1368 (7)	0.118 (4)	
H23A	0.8713	0.5043	0.0849	0.142*	
H23B	0.9277	0.4704	0.1705	0.142*	
C24	0.3253 (8)	0.6751 (6)	0.0274 (4)	0.086 (3)	
H24A	0.2850	0.7308	0.0305	0.103*	
H24B	0.3280	0.6664	−0.0246	0.103*	
C25	0.2548 (10)	0.6089 (6)	0.0486 (4)	0.094 (3)	
H25	0.2907	0.5541	0.0613	0.112*	
C26	0.1296 (8)	0.6254 (7)	0.0506 (6)	0.105 (3)	
H26A	0.0941	0.6803	0.0379	0.126*	
H26B	0.0816	0.5815	0.0647	0.126*	

### Atomic displacement parameters (Å^2^)

**Table:**

	*U*^11^	*U*^22^	*U*^33^	*U*^12^	*U*^13^	*U*^23^
Br1	0.0615 (4)	0.0512 (4)	0.0634 (5)	−0.0018 (3)	0.0158 (3)	0.0067 (3)
Br2	0.0578 (4)	0.0624 (4)	0.0460 (4)	−0.0059 (3)	0.0139 (3)	−0.0070 (3)
Br3	0.0574 (4)	0.0742 (5)	0.0679 (5)	−0.0087 (4)	0.0143 (4)	−0.0175 (4)
Br4	0.0731 (5)	0.0568 (4)	0.0580 (4)	0.0202 (3)	0.0283 (4)	0.0101 (3)
Cu1	0.0519 (5)	0.0441 (4)	0.0485 (5)	0.0020 (3)	0.0150 (4)	−0.0008 (3)
N1	0.051 (3)	0.046 (3)	0.056 (3)	−0.003 (2)	0.016 (3)	−0.006 (2)
N2	0.048 (3)	0.045 (3)	0.045 (3)	0.000 (2)	0.019 (2)	0.003 (2)
N3	0.049 (3)	0.057 (3)	0.043 (3)	−0.003 (2)	0.010 (2)	0.001 (2)
N4	0.047 (3)	0.090 (4)	0.043 (3)	−0.006 (3)	0.012 (3)	−0.004 (3)
C1	0.066 (4)	0.036 (3)	0.049 (4)	0.001 (3)	0.019 (3)	−0.001 (3)
C2	0.069 (5)	0.048 (4)	0.073 (5)	0.002 (3)	0.040 (4)	0.002 (3)
C3	0.087 (6)	0.051 (4)	0.068 (5)	0.005 (4)	0.037 (4)	0.013 (3)
C4	0.092 (6)	0.050 (4)	0.050 (4)	0.011 (4)	0.022 (4)	0.010 (3)
C5	0.065 (4)	0.051 (4)	0.058 (4)	0.002 (3)	0.017 (4)	0.004 (3)
C6	0.062 (4)	0.039 (3)	0.044 (4)	0.003 (3)	0.018 (3)	0.003 (3)
C7	0.060 (4)	0.046 (3)	0.045 (4)	0.004 (3)	0.016 (3)	−0.001 (3)
C8	0.048 (4)	0.062 (4)	0.050 (4)	0.001 (3)	0.019 (3)	0.006 (3)
C9	0.061 (4)	0.052 (4)	0.070 (5)	−0.003 (3)	0.025 (4)	0.000 (3)
C10	0.082 (6)	0.095 (7)	0.081 (6)	−0.023 (5)	0.034 (5)	−0.015 (5)
C11	0.061 (4)	0.064 (5)	0.067 (5)	−0.006 (4)	0.015 (4)	0.000 (4)
C12	0.054 (4)	0.055 (4)	0.083 (5)	−0.002 (3)	0.022 (4)	0.011 (4)
C13	0.095 (7)	0.062 (5)	0.132 (8)	0.006 (5)	0.062 (6)	0.022 (5)
C14	0.054 (4)	0.042 (3)	0.046 (4)	0.002 (3)	0.014 (3)	0.005 (3)
C15	0.073 (5)	0.059 (4)	0.045 (4)	−0.008 (4)	0.010 (4)	−0.001 (3)
C16	0.106 (7)	0.054 (4)	0.046 (4)	−0.001 (4)	0.010 (4)	−0.007 (3)
C17	0.105 (6)	0.043 (4)	0.052 (5)	0.010 (4)	0.034 (4)	0.000 (3)
C18	0.071 (5)	0.062 (4)	0.060 (5)	0.019 (4)	0.027 (4)	0.012 (3)
C19	0.060 (4)	0.051 (4)	0.046 (4)	−0.001 (3)	0.013 (3)	0.005 (3)
C20	0.050 (4)	0.068 (4)	0.038 (3)	−0.004 (3)	0.009 (3)	−0.006 (3)
C21	0.045 (4)	0.065 (4)	0.071 (5)	−0.008 (3)	0.020 (3)	0.008 (3)
C22	0.066 (5)	0.076 (6)	0.116 (7)	0.005 (4)	0.048 (5)	0.010 (5)
C23	0.070 (6)	0.089 (7)	0.184 (11)	−0.007 (6)	0.013 (7)	−0.002 (7)
C24	0.089 (7)	0.108 (7)	0.064 (5)	−0.010 (5)	0.025 (5)	−0.002 (5)
C25	0.120 (8)	0.082 (6)	0.067 (6)	−0.016 (6)	0.004 (5)	−0.015 (5)
C26	0.056 (5)	0.120 (8)	0.145 (9)	−0.019 (5)	0.039 (6)	−0.031 (7)

### Geometric parameters (Å, °)

**Table:**

Br1—Cu1	2.3999 (10)	C10—H10B	0.9300
Br2—Cu1	2.3728 (9)	C11—C12	1.477 (9)
Br3—Cu1	2.3524 (11)	C11—H11A	0.9700
Br4—Cu1	2.4074 (10)	C11—H11B	0.9700
N1—C7	1.317 (8)	C12—C13	1.308 (10)
N1—C1	1.400 (8)	C12—H12	0.9300
N1—C11	1.474 (8)	C13—H13A	0.9300
N2—C7	1.319 (8)	C13—H13B	0.9300
N2—C6	1.402 (7)	C14—C15	1.375 (9)
N2—C8	1.472 (7)	C14—C19	1.399 (9)
N3—C20	1.319 (7)	C15—C16	1.382 (10)
N3—C14	1.397 (8)	C15—H15	0.9300
N3—C21	1.465 (8)	C16—C17	1.400 (11)
N4—C20	1.323 (8)	C16—H16	0.9300
N4—C19	1.392 (8)	C17—C18	1.368 (10)
N4—C24	1.515 (10)	C17—H17	0.9300
C1—C6	1.379 (9)	C18—C19	1.389 (9)
C1—C2	1.392 (8)	C18—H18	0.9300
C2—C3	1.360 (10)	C20—H20	0.9300
C2—H2	0.9300	C21—C22	1.510 (10)
C3—C4	1.381 (10)	C21—H21A	0.9700
C3—H3	0.9300	C21—H21B	0.9700
C4—C5	1.389 (9)	C22—C23	1.226 (12)
C4—H4	0.9300	C22—H22	0.9300
C5—C6	1.388 (9)	C23—H23A	0.9300
C5—H5	0.9300	C23—H23B	0.9300
C7—H7	0.9300	C24—C25	1.388 (11)
C8—C9	1.486 (9)	C24—H24A	0.9700
C8—H8A	0.9700	C24—H24B	0.9700
C8—H8B	0.9700	C25—C26	1.393 (12)
C9—C10	1.294 (10)	C25—H25	0.9300
C9—H9	0.9300	C26—H26A	0.9300
C10—H10A	0.9300	C26—H26B	0.9300
			
Br3—Cu1—Br2	101.25 (4)	N1—C11—H11B	109.4
Br3—Cu1—Br1	127.24 (4)	C12—C11—H11B	109.4
Br2—Cu1—Br1	105.46 (4)	H11A—C11—H11B	108.0
Br3—Cu1—Br4	100.89 (4)	C13—C12—C11	123.4 (7)
Br2—Cu1—Br4	122.96 (4)	C13—C12—H12	118.3
Br1—Cu1—Br4	101.31 (4)	C11—C12—H12	118.3
C7—N1—C1	107.7 (5)	C12—C13—H13A	120.0
C7—N1—C11	126.3 (6)	C12—C13—H13B	120.0
C1—N1—C11	125.7 (6)	H13A—C13—H13B	120.0
C7—N2—C6	107.2 (5)	C15—C14—N3	130.6 (6)
C7—N2—C8	126.7 (5)	C15—C14—C19	122.7 (6)
C6—N2—C8	126.1 (5)	N3—C14—C19	106.7 (5)
C20—N3—C14	108.2 (5)	C14—C15—C16	115.1 (7)
C20—N3—C21	124.5 (5)	C14—C15—H15	122.5
C14—N3—C21	127.3 (5)	C16—C15—H15	122.5
C20—N4—C19	109.1 (6)	C15—C16—C17	123.0 (7)
C20—N4—C24	126.7 (6)	C15—C16—H16	118.5
C19—N4—C24	123.8 (6)	C17—C16—H16	118.5
C6—C1—C2	121.8 (6)	C18—C17—C16	121.3 (7)
C6—C1—N1	106.5 (5)	C18—C17—H17	119.4
C2—C1—N1	131.7 (7)	C16—C17—H17	119.4
C3—C2—C1	115.7 (7)	C17—C18—C19	116.6 (7)
C3—C2—H2	122.2	C17—C18—H18	121.7
C1—C2—H2	122.2	C19—C18—H18	121.7
C2—C3—C4	123.1 (7)	C18—C19—N4	133.2 (7)
C2—C3—H3	118.4	C18—C19—C14	121.3 (6)
C4—C3—H3	118.4	N4—C19—C14	105.5 (6)
C3—C4—C5	121.9 (7)	N3—C20—N4	110.5 (6)
C3—C4—H4	119.1	N3—C20—H20	124.7
C5—C4—H4	119.1	N4—C20—H20	124.7
C6—C5—C4	115.1 (7)	N3—C21—C22	113.5 (5)
C6—C5—H5	122.4	N3—C21—H21A	108.9
C4—C5—H5	122.4	C22—C21—H21A	108.9
C1—C6—C5	122.4 (6)	N3—C21—H21B	108.9
C1—C6—N2	106.9 (5)	C22—C21—H21B	108.9
C5—C6—N2	130.7 (6)	H21A—C21—H21B	107.7
N1—C7—N2	111.8 (6)	C23—C22—C21	126.3 (10)
N1—C7—H7	124.1	C23—C22—H22	116.8
N2—C7—H7	124.1	C21—C22—H22	116.8
N2—C8—C9	111.1 (5)	C22—C23—H23A	120.0
N2—C8—H8A	109.4	C22—C23—H23B	120.0
C9—C8—H8A	109.4	H23A—C23—H23B	120.0
N2—C8—H8B	109.4	C25—C24—N4	109.9 (8)
C9—C8—H8B	109.4	C25—C24—H24A	109.7
H8A—C8—H8B	108.0	N4—C24—H24A	109.7
C10—C9—C8	124.5 (7)	C25—C24—H24B	109.7
C10—C9—H9	117.7	N4—C24—H24B	109.7
C8—C9—H9	117.7	H24A—C24—H24B	108.2
C9—C10—H10A	120.0	C24—C25—C26	119.4 (10)
C9—C10—H10B	120.0	C24—C25—H25	120.3
H10A—C10—H10B	120.0	C26—C25—H25	120.3
N1—C11—C12	111.1 (6)	C25—C26—H26A	120.0
N1—C11—H11A	109.4	C25—C26—H26B	120.0
C12—C11—H11A	109.4	H26A—C26—H26B	120.0
